# Distribution and analysis of the resistance profiles of bacteria isolated from blood cultures in the intensive care unit

**DOI:** 10.3389/fmicb.2025.1464573

**Published:** 2025-07-14

**Authors:** Zeshi Liu, Hehui Cai, Jing Lei, Xue Zhang, Jian Yin, Yanping Zhang, Xueping Yu, Yan Geng

**Affiliations:** ^1^Department of Clinical Laboratory, The Second Affiliated Hospital of Xi’an Jiaotong University, Xi’an, Shaanxi, China; ^2^Department of Clinical Laboratory, Fujian Medical University Affiliated First Quanzhou hospital, Quanzhou, Fujian, China; ^3^Department of Infection Disease, Clinical Medical Research Center for Bacterial and Fungal Infectious Diseases of Fujian province, Fujian Medical University Affiliated First Quanzhou Hospital, Quanzhou, Fujian, China; ^4^Key Laboratory of Screening and Control of Infectious Diseases (Quanzhou Medical College), Fujian Provincial University, Quanzhou, Fujian, China

**Keywords:** blood culture, drug resistance, pathogens, intensive care unit, antimicrobial susceptibility test

## Abstract

**Purpose:**

To investigate the distribution characteristics and drug resistance of pathogenic bacteria in bloodstream infections, providing a basis for rational clinical treatment.

**Patients and methods:**

Retrospective analysis of 1,282 pathogenic strains isolated from blood cultures in the intensive care unit (ICU) of the Second Affiliated Hospital of Xi’an Jiaotong University from January 1, 2019, to December 31, 2022.

**Results:**

Gram-positive bacteria (52.0%) slightly predominated over gram-negative bacteria (48.0%). The top three gram-positive bacteria were Coagulase-negative *Staphylococcus* (28.0%), *Enterococcus faecium* (7.4%), and *Staphylococcus aureus* (6.6%). Staphylococci exhibited a high resistance rate to penicillin, oxacillin, and erythromycin; no strains resistant to vancomycin or linezolid were found. Among the Enterococci, *Enterococcus faecium* had a high resistance rate to penicillin, ampicillin, and erythromycin. Two strains of *Enterococcus faecalis* were resistant to linezolid, but none to vancomycin. The top three gram-negative bacteria were *Escherichia coli* (14.7%), *Klebsiella pneumoniae* (14.0%), and *Acinetobacter baumannii* (4.8%). The resistance rate of *Escherichia coli* to carbapenems increased from 0.0 to 2.3%. *Acinetobacter baumannii* reached 100% carbapenem resistance (up from 75.0%), while *Klebsiella pneumoniae* demonstrated 21.1-80.4% resistance to various carbapenems.

**Conclusion:**

The isolation rate of gram-positive bacteria in patients with bloodstream infection in the ICU of the Second Affiliated Hospital of Xi’an Jiaotong University was slightly higher than that of gram-negative bacteria. The alarming carbapenem resistance among gram-negative pathogens and emerging linezolid resistance in *Enterococci* demand urgent clinical interventions, including enhanced surveillance, antimicrobial stewardship, and novel therapeutic strategies.

## 1 Introduction

Bloodstream infection (BSI) is a severe systemic infectious disease characterized by the invasion of pathogenic microorganisms into the body. These microorganisms circulate in the bloodstream, where they undergo transient, intermittent, or continuous reproduction, releasing toxins and metabolic products that trigger the release of cytokines, ultimately resulting in damage to organs. In severe cases, BSI can lead to shock, multiple organ failure, disseminated intravascular coagulation, and death (Tajima et al., 2021; Fabre et al., 2022). Globally, BSIs account for an estimated 20-30% of sepsis cases and are associated with mortality rates exceeding 40% in critically ill populations, particularly in intensive care units (ICUs) (Fleischmann-Struzek et al., 2020). The rising prevalence of antimicrobial resistance (AMR) has further complicated BSI management, with the World Health Organization declaring AMR one of the top 10 global public health threats, projected to cause 10 million annual deaths by 2050 if unchecked (World Health Organization [WHO], 2021).

The diagnoses of BSIs, infective endocarditis, unexplained infections, catheter-related BSIs, arthritis, and bacterial pneumonia rely on blood culture (Gonzalez et al., 2020) to identify the causative pathogens and provide antibiotic susceptibility profiles. These data are critical for guiding evidence-based antibiotic therapy, especially as multidrug-resistant (MDR) pathogens reduce treatment efficacy and increase healthcare costs (Cheng et al., 2020; Mazi et al., 2021). Recent data reveal alarming global shifts: while gram-negative bacteria historically dominated BSI etiology, gram-positive pathogens such as *Staphylococcus aureus*, coagulase-negative staphylococci, and enterococci now prevail in many regions (Lan et al., 2021). Concurrently, resistance mechanisms like methicillin resistance in S. aureus (MRSA), vancomycin resistance in enterococci (VRE), and extended-spectrum β-lactamase (ESBL)-producing Enterobacteriaceae have escalated, driven by antibiotic overuse in clinical and agricultural settings (Antimicrobial Resistance Collaborators, 2022). For instance, MRSA accounts for > 35% of S. aureus BSIs in high-income countries, while carbapenem-resistant *Klebsiella pneumoniae* infections in ICUs exceed 60% in some endemic regions (Centers for Disease Control and Prevention [CDC], 2023; European Centre for Disease Prevention and Control [ECDC], 2022).

These trends underscore the urgency of region-specific pathogen surveillance and resistance profiling. This study retrospectively analyzed the distribution of pathogens and their antibiotic resistance in blood culture specimens collected from an intensive care unit (ICU) between 2019 and 2022. By correlating our findings with global antimicrobial resistance dynamics, we aim to establish a scientific foundation for optimizing empirical antibiotic therapy, informing stewardship programs, and mitigating resistance escalation in critical care settings.

## 2 Materials and methods

### 2.1 Materials

#### 2.1.1 Strains

Between January 1, 2019, and December 31, 2022, a total of 1,282 bacterial strains were collected from the positive blood cultures of patients in the ICU at the Second Affiliated Hospital of Xi’an Jiaotong University, with duplicate strains from the same patient excluded from the analyses. This study has been approved by the academic committee of the Stem Cell Clinical Research Institute of the Second Affiliated Hospital of Xi’an Jiaotong University and the ethics committee of the same institution (2023414). Informed consent was obtained from all study participants, and the guidelines outlined in the declaration of Helsinki were adhered to.

#### 2.1.2 Culture media and antibiotic discs

Mueller-Hinton (MH) agar (Zhengzhou AutoBio Co., Ltd., Zhengzhou, China) was used for disc-diffusion susceptibility testing, and 5% defibrinated sheep blood MH agar was used for streptococci. Culture media were sourced from AutoBio (Co., Ltd., Zhengzhou, China), and the antibiotic discs were acquired from Oxoid, Basingstoke, UK). E-test strips were obtained from Wenzhou Kangtai Biotechnology (Co., Ltd., Wenzhou, China).

### 2.2 Methods

#### 2.2.1 Bacterial identification and antimicrobial susceptibility test

The fully automated bacterial culture system BacT/ALERT 3D (Marcy l’Etoile, bioMérieux, France) was used to detect blood culture specimens. Bacterial identification and antimicrobial susceptibility tests were conducted using the VITEK 2-Compact bacterial identification system (Marcy l’Etoile, bioMérieux, France), while less common bacteria were identified using the VITEK MS system (Marcy l’Etoile, bioMérieux, France). The interpretation of antimicrobial susceptibility results followed the 2022 performance standards proposed by the Clinical and Laboratory Standards Institute (Clinical and Laboratory Standards Institute [CLSI], 2022). To exclude duplicate strains from the same patient, only the first positive blood culture with a specific pathogen per patient was included in the analysis. Subsequent isolates of the same species from the same patient within 30 days were excluded unless they exhibited distinct antimicrobial susceptibility profiles or were isolated from different anatomical sites, as recommended by international guidelines for bloodstream infection surveillance (Magiorakos et al., 2017).

#### 2.2.2 Phenotype testing for important drug resistance

Carbapenem-resistant Enterobacterales were defined as Enterobacterale specimens resistant to any of the carbapenem antibiotics, specifically imipenem, meropenem, or ertapenem.

#### 2.2.3 Quality control strains

The quality control strains used in this study included *Escherichia coli* (ATCC 25922 and ATCC 8739), *Klebsiella pneumoniae* (ATCC 700603), *Staphylococcus aureus* (ATCC 25923 and ATCC 29213), *Pseudomonas aeruginosa* (ATCC 27853), *Enterococcus faecalis* (ATCC 29212), *Streptococcus pneumoniae* (ATCC 49619), and *Haemophilus influenzae* (ATCC 49247). These strains were selected based on CLSI recommendations for antimicrobial susceptibility testing (Clinical and Laboratory Standards Institute [CLSI], 2022) and represent common pathogens associated with bloodstream infections. All strains were procured from the American Type Culture Collection (ATCC) to ensure traceability and standardized phenotypic characteristics. Quality control testing was performed weekly alongside clinical isolates, with acceptable ranges defined by CLSI criteria. No deviations from standard protocols were observed during the study period, as confirmed by internal audit records.

### 2.3 Statistical analysis

The laboratory data analysis and statistical analyses were conducted using WHONET 5.6 software (China).^1^ SPSS 24.0 software was utilized for statistical analysis. The chi-square test was employed to examine differences in categorical data.

## 3 Results

### 3.1 Bacterial distribution

Between January 2019 and December 2022, a total of 1,282 distinct pathogenic strains were isolated from the blood cultures of patients in the ICU (Table 1). Among these, 667 (52.0%) were gram-positive bacteria, while 615 (48.0%) were gram-negative bacteria. The five most prevalent bacterial species were coagulase-negative *Staphylococcus* (359 strains; 28.0%), *Escherichia coli* (189 strains; 14.7%), *Klebsiella pneumoniae* (180 strains; 14.0%), *Enterococcus faecium* (95 strains; 7.4%), and *Staphylococcus aureus* (85 strains;6.6%). Other gram-negative bacteria included *Raoultella planticola*, *Citrobacter freundii*, *Brucella*, *Haemophilus influenzae*, *Morganella morganii*, and *Citrobacter diversus*. Other streptococci mainly encompassed *Streptococcus viridans*, *Streptococcus mitis*, and *Streptococcus agalactiae*, while other gram-positive cocci included *Enterococcus gallinarum*, *Streptococcus bovis*, *Abiotrophia defectiva*, and *Gemella* species.

**TABLE 1:** Distribution of 1282 pathogenic bacteria in blood cultures.

Organism	2019(*n* = 156)		2020 (*n* = 263)		2021 (*n* = 384)		2022 (*n* = 479)		Total (*n* = 1282)	
	*n*	%	*n*	%	*n*	%	*n*	%	*N*	%
Gram-negative bacteria	98	7.6	126	9.8	173	13.5	218	17.0	615	48.0
*Escherichia coli*	24	1.9	15	1.2	64	5.0	86	6.7	189	14.7
*Klebsiella pneumoniae*	46	3.6	47	3.7	49	3.8	38	3.0	180	14.0
*Acinetobacter baumannii*	14	1.1	12	0.9	18	1.4	18	1.4	62	4.8
*Stenotrophomonas maltophilia*	0	0.0	8	0.6	13	1.0	9	0.7	30	2.3
*Pseudomonas aeruginosa*	4	0.3	13	1.0	4	0.3	24	1.9	45	3.5
*Enterobacter cloacae*	4	0.3	9	0.7	6	0.5	21	1.6	40	3.1
Klebsiella oxytoca	4	0.3	1	0.1	0	0.0	8	0.6	13	1.0
Serratia marcescens	0	0.0	0	0.0	3	0.2	6	0.5	9	0.7
Enterobacter aerogenes	0	0.0	0	0.0	5	0.4	0	0.0	5	0.4
Burkholderia cepacia	0	0.0	2	0.2	2	0.2	0	0.0	4	0.3
Other gram-negative bacteria	2	0.2	19	1.5	9	0.7	8	0.6	36	2.8
Gram -positive bacteria	58	4.5	137	10.7	211	16.5	261	20.4	667	52.0
Coagulase-negative Staphylococcus	33	2.6	53	4.1	136	10.6	137	10.7	359	28.0
*Enterococcus faecium*	7	0.5	26	2.0	34	2.7	28	2.2	95	7.4
*Staphylococcus aureus*	14	1.1	25	2.0	17	1.3	29	2.3	85	6.6
*Enterococcus faecalis*	1	0.1	7	0.5	4	0.3	10	0.8	22	1.7
*Streptococcus pneumoniae*	2	0.2	13	1.0	0	0.0	0	0.0	15	1.2
Alpha-hemolytic *Streptococcus*	1	0.1	4	0.3	4	0.3	12	0.9	21	1.6
Beta-hemolytic *Streptococcus*	0	0.0	2	0.2	0	0.0	0	0.0	2	0.2
Other gram-positive bacteria	0	0.0	7	0.5	16	1.2	45	3.5	68	5.3

### 3.2 Antibiotic resistances of major gram-positive bacteria

#### 3.2.1 *Staphylococcus* genus

A total of 443 strains of *Staphylococcus* genus were isolated, comprising 34.6% of all isolated pathogens. Among them, 85 strains were *Staphylococcus aureus*, and 358 strains were coagulase-negative staphylococci. The detection rates of methicillin-resistant *Staphylococcus aureus* (MRSA) in 2019, 2020, 2021, and 2022 were 2.3% (10 strains), 1.8% (8 strains), 0.0% (0 strains), and 2.7% (12 strains), respectively (Table 2). With the exception of 2021, the detection rates remained stable. MRSA exhibited a consistent decline in resistance to gentamicin, levofloxacin, moxifloxacin, trimethoprim-sulfamethoxazole, and erythromycin from 2019 to 2022. The resistance rate to gentamicin dropped from 75.0 to 0.0%, while the resistance rate to erythromycin decreased from 100.0 to 41.7%. Methicillin-sensitive *Staphylococcus aureus* displayed a constant resistance rate to penicillin G from 2019-2022 (100.0%), while the resistance rates to levofloxacin, moxifloxacin, and erythromycin decreased from 2019-2022. *Staphylococcus aureus* was not resistant to vancomycin, linezolid, or rifampicin.

**TABLE 2:** Resistance and sensitivity rates of *Staphylococcus aureus* isolated from blood culture to antimicrobial agents from 2019 to 2022.

Antimicrobial agent	2019				2020				2021				2022			
	MRSA (*n*=10)		MSSA (*n*=4,strains)		MRSA (*n*=8,strains)		MSSA (*n*=17)		MRSA (*n*=0)		MSSA (*n*=17)		MRSA (*n*=12)		MSSA (*n*=17)	
	R	S	R	S	R	S	R	S	R	S	R	S	R	S	R	S
Penicillin G	100.0	0.0	4	0	8	0	100.0	0.0	NA	NA	100.0	0.0	100.0	0.0	100.0	0.0
Oxacillin	100.0	0.0	0	4	8	0	0.0	100.0	NA	NA	0.0	100.0	100.0	0.0	0.0	100.0
Gentamicin	0.0	100.0	0	4	6	2	0.0	100.0	NA	NA	0.0	100.0	0.0	91.7	0.0	100.0
Rifampin	0.0	100.0	0	4	0	8	0.0	100.0	NA	NA	0.0	100.0	0.0	100.0	0.0	100.0
Levofloxacin	20.0	40.0	0	4	4	4	47.1	52.9	NA	NA	0.0	100.0	16.7	83.3	11.8	88.2
Moxifloxacin	20.0	80.0	0	4	4	4	47.1	52.9	NA	NA	0.0	100.0	8.3	83.3	11.8	88.2
Trimethoprim/sulfamethoxazole	0.0	100.0	0	4	2	6	0.0	100.0	NA	NA	11.8	88.2	0.0	100.0	11.8	88.2
Erythromycin	100.0	0.0	3	1	8	0	47.1	52.9	NA	NA	70.6	29.4	41.7	58.3	41.2	58.8
Linezolid	0.0	100.0	0	4	0	8	0.0	100.0	NA	NA	0.0	100.0	0.0	100.0	0.0	100
Vancomycin	0.0	100.0	0	4	0	8	0.0	100.0	NA	NA	0.0	100.0	0.0	100.0	0.0	100

MRSA, Methicillin-resistant *Staphylococcus aureus*; MSSA, Methicillin-sensitive *Staphylococcus aureus*; Less than 10 bacterial strains, the resistance and sensitivity rates are replaced by the number of bacterial strains; NA, not available.

Between 2019 and 2022, the detection rates of methicillin-resistant coagulase-negative Staphylococcus (MRCNS) were 7.2% (32 strains), 9.9% (44 strains), 26.2% (116 strains), and 24.8% (110 strains), respectively (Table 3). MRCNS did not show significant changes in resistance rates to gentamicin, rifampicin, levofloxacin, moxifloxacin, or erythromycin. However, the resistance rate to trimethoprim-sulfamethoxazole increased from 27.3% in 2020 to 41.8% in 2022. No instances of resistance to vancomycin or linezolid were detected among coagulase-negative Staphylococcus strains. The resistance to penicillin G and erythromycin remained relatively high among methicillin-sensitive coagulase-negative Staphylococcus strains (MSCNS). The resistance rates of MSCNS to levofloxacin, moxifloxacin, and trimethoprim-sulfamethoxazole decreased from 2019 to 2022.

**TABLE 3:** Resistance and sensitivity rates of coagulase-negative *Staphylococcus* isolated from blood culture to antimicrobial agents from 2019 to 2022.

Antimicrobial agent	MRCNS							
	2019 (*n*=25)		2020 (*n*=44)		2021 (*n*=116)		2022 (*n*=110)	
	R	S	R	S	R	S	R	S
Penicillin G	100.0	0.0	100.0	0.0	100.0	0.0	100.0	0.0
Oxacillin	100.0	0.0	100.0	0.0	100.0	0.0	100.0	0.0
Gentamicin	28.0	68.0	18.2	68.2	6.0	75.0	20.9	70.9
Rifampin	16.0	84.0	11.4	88.6	4.3	93.1	12.7	84.5
Levofloxacin	80.0	12.0	68.2	31.8	65.5	29.3	73.6	25.5
Moxifloxacin	64.0	12.0	52.3	31.8	48.3	29.3	53.6	25.5
Trime-thoprim/sulfamethoxazole	72.0	0.0	27.3	72.7	32.8	67.2	41.8	58.2
Erythromycin	96.0	4.0	81.8	13.6	84.5	13.8	74.5	25.5
Linezolid	0.0	100.0	0.0	100	0.0	100	0.0	100.0
Vancomycin	0.0	100.0	0.0	100	0.0	100	0.0	100.0
**Antimicrobial agent**	**MSCNS**							
	**2019 (*n*=6)**		**2020 (*n*=8)**		**2021(*n*=20)**		**2022(*n*=27)**	
	**R (strains)**	**S (strains)**	**R (strains)**	**S (strains)**	**R**	**S**	**R**	**S**
Penicillin G	6	0	8	0	55.0	45.0	85.2	14.8
Oxacillin	0	6	0	8	0.0	100.0	0.0	100.0
Gentamicin	0	6	0	8	0.0	90.0	0.0	100.0
Rifampin	0	6	0	8	10.0	90.0	0.0	100.0
Levofloxacin	4	2	6	2	30.0	70.0	22.2	77.8
Moxifloxacin	2	4	3	5	15.0	70.0	0.0	77.8
Trime-thoprim/Sulfamethoxazole	2	4	3	5	25.0	75.0	12.0	88.0
Erythromycin	4	3	5	3	65.0	25.0	59.3	37.0
Linezolid	0	6	0	8	0.0	100.0	0.0	100.0
Vancomycin	0	6	0	8	0.0	100.0	0.0	100.0

MRCNS, methicillin-resistant coagulase-negative *Staphylococcus*; MSCNS, methicillin-sensitive coagulase-negative *Staphylococcus*. Less than 10 bacterial strains, the resistance and sensitivity rates are replaced by the number of bacterial strains.

#### 3.2.2 *Enterococcus* genus

A total of 132 strains from the Enterococcus genus were isolated, including 22 strains of *Enterococcus faecalis*, 95 strains of *Enterococcus faecium*, and 15 strains of other Enterococcus species. *Enterococcus faecium* exhibited > 80.0% resistance to ampicillin, though *Enterococcus faecalis* was sensitive to the drug (Table 4). *Enterococcus faecium* displayed significantly higher antibiotic resistance rates than those displayed by *Enterococcus faecalis*. *Enterococcus faecium* demonstrated a resistance rate > 90% to penicillin, although its resistance rate to erythromycin declined from 2019-2022. In contrast, *Enterococcus faecalis* exhibited resistance rates of 20.0% and 40.0% to penicillin and erythromycin, respectively. Neither *Enterococcus faecium* nor *Enterococcus faecalis* were resistant to vancomycin, though two strains of *Enterococcus faecium* were found to be resistant to linezolid.

**TABLE 4:** Resistance and sensitivity rates of *Enterococcus* spp. isolated from blood culture to antimicrobial agents from 2019 to 2022.

Antimicrobial agent	Entercocccus faecium							
	2019 (*n*=1)		2020 (*n*=26)		2021 (*n*=34)		2022 (*n*=28)	
	R(strains)	S(strains)	R	S	R	S	R	S
Penicillin G	0	1	100.0	0.0	91.2	8.8	100.0	0.0
Ampicillin	0	1	100.0	0.0	88.2	11.8	100.0	0.0
Gentamicin-high	0	1	34.6	61.5	32.4	67.6	39.3	60.7
Erythromycin	1	0	96.2	0.0	82.4	0.0	50.0	50.0
Levofloxacin	1	0	69.2	30.8	64.7	29.4	55.9	28.6
Linezolid	0	1	0.0	100.0	0.0	100.0	0.0	100.0
Vancomycin	0	1	0.0	100.0	0.0	100.0	0.0	100.0
**Antimicrobial agent**	**Entercocccus faecalis**							
	**2019 (*n*=7)**		**2020 (*n*=7)**		**2021 (*n*=4)**		**2022 (*n*=10)**	
	**R (strains)**	**S (strains)**	**R (strains)**	**S (strains)**	**R (strains)**	**S (strains)**	**R**	**S**
Penicillin G	3	4	3	4	2	2	20.0	80.0
Ampicillin	0	7	0	7	0	4	0.0	100.0
Gentamicin-high	3	4	2	5	1	3	20.0	80.0
Erythromycin	7	0	7	0	3	1	40.0	0.0
Levofloxacin	2	4	1	6	1	3	10.0	90.0
Linezolid	0	7	0	7	0	4	20.0	80.0
Vancomycin	0	7	0	7	0	4	0.0	100.0

Less than 10 bacterial strains, the resistance and sensitivity rates are replaced by the number of bacterial strains.

### 3.3 Antibiotic resistances of enterobacterales

#### 3.3.1 *Escherichia coli*

*Escherichia coli* exhibited increased carbapenem resistance rising from 0.0% in 2019 to 2.3% in 2022 (Table 5). The resistance rates of *Escherichia coli* to cefazolin, ceftazidime, and ceftriaxone exceeded 20.0%, with resistance to cefazolin and ceftriaxone increasing each year. However, resistance rates to amikacin and piperacillin-tazobactam remained relatively low. No strains were found resistant to imipenem, meropenem, tigecycline, or polymyxin B. Resistance to ampicillin-Sulbactam, Cefazolin, Cefepime, Levofloxacin, and Trime-thoprim/sulfamethoxazole varied significantly each year, demonstrating statistical significance.

**TABLE 5:** Resistance and sensitivity rates of *Escherichia Coli* isolated from blood culture to antimicrobial agents from 2019 to 2022.

Antimicrobial agent	2019(*n*=24)		2020 (*n*=15)		2021 (*n*=64)		2022 (*n*=86)		χ^2^	*P*
	R	S	R	S	R	S	R	S		
Ampicillin	79.2	20.8	93.3	6.7	79.7	12.5	90.7	9.3	5.194	0.158
Piperacillin	54.2	45.8	60.0	33.3	78.1	20.3	74.4	19.8	6.292	0.098
Ampicillin-Sulbactam	33.3	50.0	80.0	6.7	64.1	28.1	59.3	25.6	9.966	0.019
Piperacillin-Tazobactam	0.0	100.0	0.0	100.0	1.6	96.9	4.7	94.2	2.694	0.441
Cefazolin	45.8	54.2	46.7	53.3	87.5	12.5	69.7	30.2	20.180	< 0.001
Ceftazidime	26.3	73.7	26.7	73.3	32.8	65.6	32.6	67.4	0.725	0.867
Ceftriaxone	58.3	41.7	46.7	53.3	60.9	39.1	58.1	41.9	1.018	0.797
Cefepime	41.7	50.0	35.8	60.3	34.4	59.4	7.0	86.0	22.938	< 0.001
Cefotetan	0.0	100.0	0.0	100.0	0	100	2.3	96.5	–	–
Aztreonam	33.3	66.7	46.7	53.3	48.4	51.6	37.2	62.8	2.746	0.433
Imipenem	0.0	100.0	0.0	100.0	0	100	2.3	97.7	–	–
Meropenem	0.0	100.0	0.0	100.0	0	100	2.3	97.7	–	–
Amikacin	0.0	100.0	0.0	100.0	1.6	98.4	0.0	100	–	–
Gentamicin	20.8	79.2	33.3	66.7	43.8	53.1	37.2	62.8	4.028	0.258
Tobramycin	16.7	66.7	20.0	66.7	21.9	50.0	15.1	67.4	1.344	0.726
Ciprofloxacin	45.8	54.2	73.3	26.7	51.6	45.3	62.8	37.2	4.787	0.188
Levofloxacin	25.0	66.7	73.3	26.7	51.6	48.4	60.5	33.7	11.982	0.007
Trime-thoprim/sulfamethoxazole	33.3	66.7	26.7	73.3	56.2	43.8	55.8	44.2	8.051	0.045
Tigecycline	0.0	100.0	0.0	100.0	0.0	100	0.0	100	–	–
Polymyxin B	0.0	100.0	0.0	100.0	0.0	100	0.0	100	–	–

#### 3.3.2 *Klebsiella pneumoniae*

*Klebsiella pneumoniae* demonstrated resistance rates to carbapenem antibiotics that were consistently exceeding 20.0% from 2019 to 2022 (Table 6). The resistance rates of cefazolin, ceftazidime, ceftriaxone, and cefepime exceeded 20.0%. *Klebsiella pneumoniae* demonstrated slightly higher resistance rates than *Escherichia coli*. Both bacteria exhibited their highest resistance rates in 2021, with some declines observed in 2022. Significant annual variations in resistance rates were observed for all tested antibiotics (*p* < 0.05).

**TABLE 6:** Resistance and sensitivity rates of *Klebsiella pneumoniae* isolated from blood culture to antimicrobial agents from 2019 to 2022.

Antimicrobial agent	2019(n = 46)		2020 (*n* = 47)		2021 (*n* = 49)		2022 (*n* = 38)		χ^2^	*P*
	R	S	R	S	R	S	R	S		
Piperacillin	23.4	76.6	38.3	61.7	55.1	22.4	15.8	81.6	17.685	0.001
Ampicillin-Sulbactam	24.5	72.7	59.6	40.4	79.6	20.4	14.4	83.2	45.287	< 0.001
Piperacillin-Tazobactam	20.2	77.8	29.8	70.2	49.0	49.0	11.1	88.9	16.170	0.001
Cefazolin	50.2	48.7	53.2	44.8	81.6	17.4	53.2	41.1	13.947	0.003
Ceftazidime	24.9	69.1	38.3	53.2	59.2	36.7	23.7	76.3	13.695	0.003
Ceftriaxone	23.1	68.9	40.4	58.6	65.3	34.7	26.3	73.7	18.130	<0.001
Cefepime	22.4	72.6	29.8	70.2	59.2	40.8	21.1	73.7	17.973	<0.001
Cefotetan	28.1	71.7	19.1	63.8	14.3	85.7	2.6	97.4	10.250	0.017
Aztreonam	28.7	71.3	38.3	61.7	63.3	36.7	28.9	71.1	13.317	0.004
Imipenem	19.6	80.4	29.8	70.2	49.0	51.0	21.1	78.9	10.480	0.015
Meropenem	19.6	80.4	29.8	70.2	49.0	51.0	21.1	78.9	10.480	0.015
Amikacin	19.6	80.4	29.8	70.2	44.9	55.1	13.2	86.8	11.357	0.010
Gentamicin	15.2	84.8	29.3	70.7	40.8	59.2	10.5	89.5	11.905	0.008
Tobramycin	19.6	80.4	38.3	61.7	44.9	40.8	31.6	60.5	7.408	0.060
Ciprofloxacin	10.9	89.1	38.3	61.7	69.4	26.5	55.3	44.7	44.249	<0.001
Levofloxacin	10.9	89.1	38.3	61.7	65.3	30.6	52.6	44.7	39.089	<0.001
Trimethoprim/sulfamethoxazole	0.0	93.5	25.5	74.5	59.2	40.8	52.6	47.4	55.966	<0.001
Tigecycline	0.0	100.0	0.0	100.0	0.0	100.0	0.0	100	–	–
Polymyxin B	0.0	100.0	0.0	100.0	0.0	100.0	0.0	100	–	–

**FIGURE 1:**
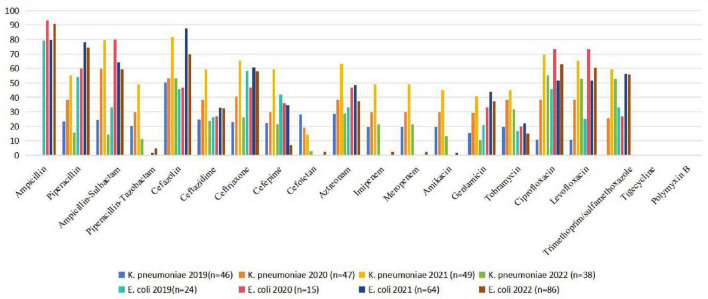
Comparison of antibiotic resistance rates of *E. coli* and *K. pneumoniae* (2019–2022). *E. coli, Escherichia coli*; *K. pneumoniae, Klebsiella pneumoniae*.

The comparison of antibiotic resistance rates between *E. coli* and *K. pneumoniae* from 2019 to 2022 is shown in Figure 1.

#### 3.3.3 *Enterobacter cloacae*

*Enterobacter cloacae* exhibited a rising trend in resistance rates to carbapenem antibiotics, increasing from 0.0% in 2019 to 19.0% in 2022 (Table 7). The resistance rates to piperacillin, cefotiam, and ceftriaxone all exceeded 80.0%. The resistance rate to piperacillin-tazobactam decreased from 75.0 to 38.1%, and the resistance rate to amikacin decreased from 100 to 52.4%. There were no significant changes in resistance rates to gentamicin, levofloxacin, and ciprofloxacin. However, the resistance rate to trimethoprim-sulfamethoxazole increased from 25.0 to 61.9%.

**TABLE 7:** Resistance and sensitivity rates of *Enterobacter cloacae* isolated from blood culture to antimicrobial agents from 2019 to 2022.

Antimicrobial agent	2019 (*n*=4)		2020 (*n*=9)		2021 (*n*=6)		2022 (*n*=21)	
	R(strains)	S (strains)	R (strains)	S (strains)	R (strains)	S (strains)	R	S
Piperacillin	4	0	9	0	6	0	85.7	0.0
Piperacillin-tazobactam	3	1	6	3	0	6	38.1	61.9
Ceftazidime	4	0	9	0	6	0	90.5	9.5
Ceftriaxone	4	0	9	0	6	0	100.0	0.0
Cefepime	1	3	3	6	0	6	33.3	47.6
Aztreonam	4	0	9	0	0	6	52.4	28.6
Imipenem	0	4	0	9	0	6	19.0	81.0
Meropenem	0	4	0	9	0	6	19.0	81.0
Amikacin	0	4	0	9	0	6	0.0	100.0
Gentamicin	1	3	3	6	0	6	47.6	52.4
Tobramycin	1	3	3	6	0	6	14.3	66.7
Ciprofloxacin	1	3	3	6	0	6	42.9	57.1
Levofloxacin	1	3	3	6	0	6	42.9	57.1
Trime-thoprim/sulfamethoxazole	1	3	3	6	0	6	61.9	38.1
Tigecycline	0	4	0	9	0	6	0.0	100.0
Polymyxin B	0	4	0	9	0	6	0.0	100.0

Less than 10 bacterial strains, the resistance and sensitivity rates are replaced by the number of bacterial strains.

### 3.4 Antibiotic resistances of non-fermenting gram-negative bacteria

#### 3.4.1 *Pseudomonas aeruginosa*

The resistance rates of *Pseudomonas aeruginosa* to imipenem and meropenem ranged from 73.7 to 84.6%, with a noticeable decrease from 2019 to 2022 (Table 8). The resistance rates to piperacillin-tazobactam, cefotiam, cefepime, amikacin, and tobramycin were steady at approximately 10.0%. The resistance rate to ciprofloxacin decreased from 84.6% in 2019 to 0.0% in 2022, as did the resistance rate to levofloxacin, which decreased from 84.6% in 2019 to 29.2% in 2022. *Pseudomonas aeruginosa* did not display any resistance to polymyxin B.

**TABLE 8:** Resistance and sensitivity rates of *Pseudomonas aeruginosa* isolated from blood culture to antimicrobial agents from 2019 to 2022.

Antimicrobial agent	2019 (*n* = 4)		2020 (*n* = 13)		2021 (*n* = 4)		2022 (*n* = 24)	
	R(strains)	S(strains)	R(strains)	S(strains)	R(strains)	S(strains)	R	S
Piperacillin-tazobactam	0	4	15.4	69.2	0	4	12.5	70.8
Ceftazidime	0	4	7.7	76.9	0	4	10.0	79.2
Cefepime	0	4	7.7	84.6	0	4	8.3	83.3
Imipenem	0	4	84.6	15.4	0	4	75.0	20.8
Meropenem	0	4	84.6	15.4	0	4	70.8	29.2
Amikacin	0	4	0.0	100	0	4	4.2	95.8
Tobramycin	0	4	7.7	92.3	0	4	0.0	100
Ciprofloxacin	2	2	84.6	15.4	2	2	0.0	100
Levofloxacin	2	2	84.6	15.4	2	2	29.2	62.5
Polymyxin B	0	4	0.0	100.0	0	4	0.0	100.0

Less than 10 bacterial strains, the resistance and sensitivity rates are replaced by the number of bacterial strains.

#### 3.4.2 *Acinetobacter baumannii*

*Acinetobacter baumannii* displayed a notable resistance to multiple antibiotics (Table 9). The resistance rate to amikacin increased from 16.7 to 72.2% in 2022, while resistance rates to imipenem and meropenem were consistently > 75.0%. The resistance rates to other antibiotics also exhibited a rising trend from 2019 to 2022. *Acinetobacter baumannii* did not display any resistance to tigecycline or polymyxin B. Resistance to ampicillin-Sulbactam, piperacillin-tazobactam, imipenem, meropenem, amikacin, tobramycin and Trime-thoprim/sulfamethoxazole varied significantly each year, demonstrating statistical significance (*p* < 0.05).

**TABLE 9:** Resistance and sensitivity rates of *Acinetobacter baumannii* isolated from blood culture to antimicrobial agents from 2019 to 2022.

Antimicrobial agent	2019 (*n*=14)		2020 (*n*=12)		2021 (*n*=18)		2022 (*n*=18)		χ^2^/*F*	*P*
	R	S	R	S	R	S	R	S		
Piperacillin	100.0	0.0	100.0	0.0	100	0.0	100.0	0.0	–	–
Ampicillin/Sulbactam	42.9	57.1	58.3	25.0	72.2	11.1	77.8	5.6	13.328	0.021
Piperacillin-tazobactam	92.9	7.1	75.0	25.0	100	0.0	100.0	0.0	9.528	0.014
Ceftazidime	92.9	7.1	66.7	16.7	83.3	0.0	88.9	0.0	6.949	0.243
Cefepime	92.9	7.1	66.7	25.0	77.8	0.0	83.3	0.0	9.719	0.056
Imipenem	92.9	7.1	75.0	25.0	100	0.0	100.0	0.0	6.530	0.019
Meropenem	92.9	7.1	75.0	25.0	100	0.0	100.0	0.0	6.530	0.019
Amikacin	42.9	57.1	16.7	83.3	55.6	38.9	72.2	22.2	35.209	< 0.001
Gentamicin	42.9	57.1	50.0	33.3	66.7	16.7	77.8	11.1	10.674	0.069
Tobramycin	42.9	57.1	75.0	25.0	72.2	11.1	100.0	0.0	19.706	< 0.001
Ciprofloxacin	57.1	42.9	58.3	41.7	61.1	16.7	88.9	5.6	12.554	0.023
Levofloxacin	50.0	50.0	50.0	50.0	66.7	22.2	66.7	16.7	8.076	0.180
Trime-thoprim/sulfamethoxazole	21.4	78.6	33.3	66.7	55.6	44.4	83.3	16.7	14.186	0.002
Tigecycline	0.0	100.0	0.0	100.0	0.0	100.0	0.0	100.0	-	-
Polymyxin B	0.0	100.0	0.0	100.0	0.0	100.0	0.0	100.0	-	-

### 3.5 Carbapenem-resistant gram-negative bacilli

Between 2019 and 2022, the detection rates of carbapenem-resistant organisms (CRO) among gram-negative bacilli varied (Table 10). In 2019, CRO accounted for 4.1% (52 strains) of gram-negative bacilli, including 37 strains of *Klebsiella pneumoniae*, 2 strains of *Pseudomonas aeruginosa*, and 13 strains of *Acinetobacter baumannii*. In 2020, CRO accounted for 3.1% (40 strains) of gram-negative bacilli, including 14 strains of *Klebsiella pneumoniae*, 6 strains of *Raoultella planticola*, 11 strains of *Pseudomonas aeruginosa*, and 9 strains of *Acinetobacter baumannii*. In 2021, the CRO detection rate was 3.3% (42 strains), including 24 strains of *Klebsiella pneumoniae* and 18 strains of *Acinetobacter baumannii*. In 2022, the CRO detection was 3.8% (49 strains), including 2 strains of *Escherichia coli*, 8 strains of *Klebsiella pneumoniae*, 4 strains of *Enterobacter cloacae*, 17 strains of *Pseudomonas aeruginosa*, and 18 strains of *Acinetobacter baumannii*. Carbapenem-resistant *Klebsiella pneumoniae* (CRKP) and Carbapenem-resistant *Acinetobacter baumannii* (CRAB) were consistently identified from 2019 to 2022. CRKP showed a decreasing resistance to aminoglycosides but an increasing resistance to trimethoprim-sulfamethoxazole. Conversely, CRAB exhibited increasing resistance rates to amikacin and gentamicin, from 16.7% in 2019 to 77.8% in 2022 and from 66.7% in 2019 to 94.4% in 2022, respectively. The resistance to trimethoprim-sulfamethoxazole increased from 61.5% in 2019 to 69.1% in 2022, while resistance to levofloxacin decreased from 100.0% in 2019 to 66.7% in 2022. Carbapenem-resistant *Enterobacter cloacae* (CREC) was only detected in 2022 and was sensitive to tigecycline, polymyxin B, and aminoglycoside antibiotics. Carbapenem-resistant *Pseudomonas aeruginosa* (CRPA) was identified in 2019, 2020, and 2022, with resistance rates to ceftazidime and cefepime increasing from 0.0% in 2019 to 70.6% in 2022 and from 0.0% in 2019 to 52.7% in 2022, respectively. The resistance rates of CRPA to ciprofloxacin and levofloxacin decreased from 100.0% in 2019 to 41.2% and 52.9% in 2022, respectively.

**TABLE 10:** Resistance rate and sensitivity rate of carbapenem resistant gram-negative bacteria isolated from blood culture to antimicrobial agents from 2019 to 2022.

Antimicrobial agents	2020(*n*=40)								2021(*n*=42)				2022(*n*=49)									
	kpn(*n* = 14)		kpl(*n*=6)		pae(*n*=11)		aba(*n*=9)		kpn(*n*=24)		aba(*n*=18)		eco(*n*=2)		kpn(*n*=8)		ecl(*n*=4)		pae(*n*=17)		aba(*n*=18)	
	R	S	R	S	R	S	R	S	R	S	R	S	R	S	R	S	R	S	R	S	R	S
Ampicillin	NA	NA	NA	NA	NA	NA	NA	NA	NA	NA	NA	NA	100.0	0.0	NA	NA	NA	NA	NA	NA	NA	NA
Piperacillin	100.0	0.0	100.0	0.0	NA	NA	100.0	0.0	100.0	0.0	100.0	0.0	100.0	0.0	100.0	0.0	NA	NA	NA	NA	100.0	0
Ampicillin/Sulbactam	100.0	0.0	100.0	0.0	NA	NA	100.0	0.0	100.0	0.0	100.0	0.0	100.0	0.0	100.0	0.0	NA	NA	NA	NA	100.0	0
Piperacillin-tazobactam	100.0	0.0	100.0	0.0	0.0	90.9	100.0	0.0	100.0	0.0	100.0	0.0	100.0	0.0	100.0	0.0	100.0	0.0	0.0	100.0	100.0	0
Cefazolin	100.0	0.0	100.0	0.0	NA	NA	NA	NA	100.0	0.0	NA	NA	100.0	0.0	100.0	0.0	NA	NA	NA	NA	NA	NA
Ceftazidime	100.0	0.0	100.0	0.0	0.0	81.8	100.0	0.0	100.0	0.0	100.0	0.0	100.0	0.0	100.0	0.0	100.0	0.0	70.6	11.8	100.0	0
Ceftriaxone	100.0	0.0	100.0	0.0	NA	NA	100.0	0.0	100.0	0.0	100.0	0.0	100.0	0.0	100.0	0.0	100.0	0.0	NA	NA	100.0	0
Cefepime	100.0	0.0	100.0	0.0	0.0	90.9	100.0	0.0	100.0	0.0	100.0	0.0	100.0	0.0	100.0	0.0	100.0	0.0	52.9	29.4	100.0	0
Cefotetan	100.0	0.0	100.0	0.0	NA	NA	NA	NA	100.0	0.0	NA	NA	100.0	0.0	100.0	0.0	NA	NA	NA	NA	NA	NA
Aztreonam	100.0	0.0	0.0	100.0	NA	NA	NA	NA	100.0	0.0	NA	NA	100.0	0.0	100.0	0.0	0.0	100.0	NA	NA	NA	NA
Imipenem	100.0	0.0	100.0	0.0	100.0	0.0	100.0	0.0	100.0	0.0	100.0	0.0	100.0	0.0	100.0	0.0	100.0	0.0	100.0	0.0	100.0	0
Meropenem	100.0	0.0	100.0	0.0	100.0	0.0	100.0	0.0	100.0	0.0	100.0	0.0	100.0	0.0	100.0	0.0	100.0	0.0	88.2	11.8	100.0	0
Amikacin	100.0	0.0	0.0	100.0	0.0	100.0	22.2	77.8	83.3	16.7	16.7	83.3	0.0	100.0	50.0	50.0	0.0	100.0	0.0	100.0	77.8	22.2
Gentamicin	100.0	0.0	100.0	0.0	NA	NA	66.7	11.1	100.0	0.0	66.7	33.3	0.0	100.0	62.5	37.5	0.0	100.0	NA	NA	94.4	0
Tobramycin	100.0	0.0	100.0	0.0	9.1	90.9	100.0	0.0	83.3	16.7	100.0	0.0	0.0	100.0	50.0	50.0	0.0	100.0	0.0	100.0	100.0	0
Ciprofloxacin	100.0	0.0	100.0	0.0	100.0	0.0	100.0	0.0	100.0	0.0	100.0	0.0	100.0	0.0	100.0	0.0	0.0	100.0	41.2	47.1	100.0	0
Levofloxacin	100.0	0.0	100.0	0.0	100.0	0.0	100.0	0.0	100.0	0.0	83.3	11.1	100.0	0.0	100.0	0.0	0.0	100.0	52.9	23.5	66.7	33.3
Trime-thoprim/sulfamethoxazole	28.6	71.4	100.0	0.0	NA	NA	44.4	55.6	100.0	0.0	61.1	38.9	100.0	0.0	62.5	37.5	100.0	0.0	NA	NA	77.8	22.2
Tigecycline	0.0	100.0	0.0	100.0	NA	NA	0.0	100.0	0.0	100.0	0.0	100.0	0.0	100.0	0.0	100.0	0.0	100.0	NA	NA	0.0	100.0
Polymyxin B	0.0	100.0	0.0	100.0	0.0	100.0	0.0	100.0	0.0	100.0	0.0	100.0	0.0	100.0	0.0	100.0	0.0	100.0	0.0	100.0	0.0	100.0

Kpn, *Klebsiella pneumoniae*; kpl, *Raoultella planticola*; pae, *Pseudomonas aeruginosa*; aba, *Acinetobacter baumannii*; eco, *Escherichia coli*; ecl, *Enterobacter cloacae*; NA, not available.

## 4 Discussion

Blood culture remains the gold standard for diagnosing bloodstream infections (BSIs) due to its accessibility, clinical utility, and ability to guide antibiotic susceptibility testing (Bai et al., 2022). While emerging molecular diagnostics (e.g., PCR-based assays) offer rapid pathogen identification, blood culture remains indispensable for capturing viable pathogens and resistance profiles, particularly in critically ill ICU patients requiring timely targeted therapy (El Haddad et al., 2018). The isolation of clinically-relevant pathogens via blood culture indicates that the defense mechanisms of the host and/or prior clinical interventions were unsuccessful in eradicating the infecting pathogens at the primary infection site. Moreover, the specific types of pathogens identified via blood culture offer important prognostic insights (El Haddad et al., 2018; Wildenthal et al., 2023). When multidrug-resistant organisms are identified in blood cultures, the patient mortality rate is as high as 35% (Abu-Saleh et al., 2018; GBD 2019 Antimicrobial Resistance Collaborators, 2022).

From 2019 to 2022, a total of 1,282 distinct strains were isolated from the positive blood cultures of patients in the ICU at the Second Affiliated Hospital of Xi’an Jiaotong University. gram-positive bacteria (52.0%) slightly outnumbered gram-negative isolates (48.0%), aligning with global ICU trends (Li et al., 2022; Van An et al., 2023). However, the data in this study may be biased due to a lower number of strains in 2021. Among the gram-positive bacteria, coagulase-negative staphylococci were the most common, followed by *Enterococcus faecalis* and *Staphylococcus aureus*. MRSA displayed a decreasing resistance to gentamicin, levofloxacin, moxifloxacin, trimethoprim-sulfamethoxazole, and erythromycin from 2019 to 2022. No resistance to vancomycin, linezolid, or rifampicin was observed in *Staphylococcus aureus*. However, coagulase-negative staphylococci (CoNS) accounted for 28.0% of isolates, raising questions about their clinical significance. While CoNS are frequent blood culture contaminants due to improper skin disinfection (Wang et al., 2023), they may represent true pathogens in immunocompromised patients or those with indwelling devices (Heilmann et al., 2019). In our cohort, standardized blood culture collection were followed, yet persistent CoNS isolation underscores the need for rigorous clinical correlation to distinguish contamination from true infection. The resistance rates of MRCNS to gentamicin, rifampicin, levofloxacin, moxifloxacin, and erythromycin did not change significantly throughout the study period, though the resistance of MRCNS to trimethoprim-sulfamethoxazole increased slightly. MRCNS were not resistant to vancomycin or linezolid; therefore, these are the preferred antibiotics for clinical Staphylococcus infections. The Enterococcus genus constituted 11.0% of the isolated strains in our hospital, with *Enterococcus faecium* displaying higher resistance rates than *Enterococcus faecalis*. More specifically, *Enterococcus faecium* exhibited high resistance to ampicillin, while *Enterococcus faecalis* was sensitive to ampicillin. The resistance of *Enterococcus faecium* decreased from 2019 to 2022, while *Enterococcus faecalis* was slightly resistant to penicillin and erythromycin. Neither *Enterococcus faecium* nor *Enterococcus faecalis* displayed resistance to vancomycin, though two *Enterococcus faecium* strains were resistant to linezolid.

The rising carbapenem resistance in Enterobacterales is alarming. *Escherichia coli* exhibited a carbapenem resistance increase from 0% (2019) to 2.3% (2022), while *Klebsiella pneumoniae* maintained resistance rates exceeding 20% throughout the study period. Nevertheless, the overall resistance rates decreased from 2019 to 2022. According to the 2023 CHINET China Bacterial Resistance Surveillance data, *Klebsiella pneumoniae* had resistance rates of 26.2 and 27.1% to imipenem and meropenem, respectively, which are similar to the national resistance levels (Hu et al., 2022). These trends are likely driven by horizontal gene transfer of blaKPC carbapenemases (Han et al., 2021) and prolonged carbapenem use in critically ill patients. *Enterobacter cloacae* demonstrated an increase in resistance rates to carbapenem antibiotics from 2019 to 2022. No resistance to tigecycline or polymyxin B was observed among bacteria in Enterobacterales. Therefore, it is imperative to prioritize the identification and management of risk factors associated with carbapenemase-induced nosocomial infections.

Among non-fermenting gram-negative bacteria, *Pseudomonas aeruginosa* displayed relatively high sensitivity to piperacillin-tazobactam, cefotiam, cefepime, amikacin, and tobramycin. However, its resistance rates to imipenem and meropenem decreased during the study period. *Pseudomonas aeruginosa* did not display resistance to polymyxin B. In contrast, *Acinetobacter baumannii* remained sensitive to tigecycline and polymyxin B. However, resistance rates to imipenem and meropenem were high from 2019 to 2022. The rise in carbapenem resistance aligns with global trends (GBD 2019 Antimicrobial Resistance Collaborators, 2022), particularly in A. baumannii (100% resistance in 2022), surpassing national averages reported by CHINET (26od.%) (Hu et al., 2022). It is likely due to various factors, including compromised immunity in patients in the ICU and prolonged use of broad-spectrum antibiotics (Teerawattanapong et al., 2018; Kaye et al., 2023). This phenomenon may also be linked to the production of carbapenemase hydrolytic enzymes, decreased outer membrane permeability or loss of porins, reduced affinity of penicillin-binding proteins, and overexpression of efflux pumps (Abdi et al., 2020; Somily et al., 2022).

The ICU is a high-incidence area of multidrug-resistant organisms and carries a considerable disease burden. Research shows that the six leading pathogens for deaths associated with resistance-related deaths—*Escherichia coli*, *Staphylococcus aureus*, *Klebsiella pneumoniae*, *Streptococcus pneumoniae*, *Acinetobacter baumannii*, and *Pseudomonas aeruginosa*—were responsible for approximately 929,000 (660,000–1,270,000) deaths ascribed to AMR and 3.57 million (2.62–4.78) deaths associated with AMR in 2019. Due to various factors, the number of detected strains in individual years is relatively small, and some drug resistance rates may be skewed. Clinical implications of resistance patterns demand urgent action. For gram-positive infections, vancomycin and linezolid remain effective against *staphylococci* and *enterococci*, though two *Enterococcus faecium* linezolid-resistant strains highlight emerging threats. For gram-negative infections, carbapenem-sparing regimens (e.g., ceftazidime-avibactam for *K. pneumoniae*) should be prioritized where susceptibility permits, while polymyxins and tigecycline serve as last-resort options. For critically ill patients in the ICU, who often undergo invasive medical procedures and have multiple underlying conditions and compromised immune function, the detection of CRO in blood specimens is associated with an increased risk of mortality, emphasizing the need for prompt, effective, and precise treatment (Yi and Kim, 2021; Martínez et al., 2023).

Study limitations include its single-center, retrospective design and small annual sample sizes (e.g., 2021), which may skew resistance rates due to stochastic variation. To mitigate this, we aggregated data across the 4-year period to identify overarching trends. Additionally, infection control measures (e.g., enhanced environmental decontamination, antimicrobial stewardship programs) were implemented during the study period, potentially influencing resistance dynamics. Future multicenter studies with larger cohorts are needed to validate these findings.

## 5 Conclusion

The pathogens responsible for BSI and their antimicrobial resistance profiles are constantly changing. Timely surveillance of pathogen distribution and resistance trends in blood cultures remain indispensable for guiding empirical antibiotic choices in ICU patients with infections. The resistance patterns reported here offer actionable insights to optimize treatment regimens and inform antimicrobial stewardship efforts in critical care settings.

## Data Availability

The original contributions presented in the study are included in the article/supplementary material, further inquiries can be directed to the corresponding authors.

## Footnotes

1
www.whonet.org.cn
